# Exploring patient and NHS staff acceptability of artificial intelligence-supported surgical wound monitoring within the WISDOM feasibility study: a multi-centre qualitative interview study

**DOI:** 10.1136/bmjopen-2026-116323

**Published:** 2026-07-31

**Authors:** Judith Tanner, Melissa Rochon, Karen Cariaga, Roy Harris, Jacqueline Beckhelling, Janet Bouttell, Sarah Bolton, Keith Wilson, James Jurkiewicz, Luxmi Dhoonmoon, Nada Mostafa, Jon Dummer, Kristia Basilio, Rosalie Magboo, Kathryn Procter, Claire McMillan

**Affiliations:** 1 School of Health Sciences, University of Nottingham, Nottingham, UK; 2 Infection Prevention and Control, Guy's and St Thomas’ NHS Foundation Trust, London, UK; 3 Guy's and St Thomas’ NHS Foundation Trust, London, UK; 4 Research Support Service, University of Nottingham, Nottingham, UK; 5 University Hospitals of Derby and Burton NHS Foundation Trust, Derby, UK; 6 Centre for Healthcare Equipment and Technology, Nottingham, UK; 7 Research, Liverpool Heart and Chest Hospital NHS Foundation Trust, Liverpool, Liverpool, UK; 8 Isla Health, London, UK; 9 Central and North West London NHS Foundation Trust, London, London, UK; 10 Health Innovation East Midlands, Nottingham, UK; 11 Barts Health NHS Trust, London, UK; 12 Saint Bartholomew’s Hospital Barts Heart Centre, London, UK; 13 Newcastle Upon Tyne Hospitals NHS Foundation Trust, Newcastle upon Tyne, UK

**Keywords:** Artificial Intelligence, Digital Technology, QUALITATIVE RESEARCH, WOUND MANAGEMENT

## Abstract

**Objective:**

Artificial intelligence (AI) is beginning to be used within digital surgical wound monitoring to facilitate implementation at scale. AI acceptability is essential to its success. This study aimed to explore patient and staff acceptability of an AI-based digital surgical wound monitoring platform.

**Design:**

Qualitative interviews

**Setting:**

Two hospitals performing cardiac surgery in England.

**Participants:**

20 patients undergoing cardiac surgery and 10 clinical staff participating in a randomised feasibility trial of surgical wound monitoring with AI.

**Interventions:**

Semi-structured interviews were conducted focusing on participants’ experiences or perceptions of AI-based digital surgical wound monitoring. Data were analysed, guided by the theoretical framework of acceptability.

**Primary measure:**

Patient and staff acceptability of AI within surgical wound monitoring.

**Results:**

Patients and staff were supportive of the use of AI within surgical wound monitoring and felt safety was improved, access was increased and efficiency was improved. AI monitoring was perceived to allow the early detection and treatment of surgical wound complications and facilitate the implementation of monitoring at scale for all patients. Some participants were concerned about data security risks, staff over-reliance on AI and the loss of human interaction.

**Conclusion:**

AI-supported digital surgical wound monitoring appeared acceptable to participants within this feasibility study. Further research is needed to evaluate implementation in broader settings.

**Trial registration number:**

ISRCTN16900119 and NCT06475703.

**STRENGTHS AND LIMITATIONS OF THIS STUDY**

- This study provides data from patients and staff regarding their acceptability of artificial intelligence (AI) within digital surgical wound monitoring.
- The qualitative approach allowed for in-depth data collection and flexibility to explore new areas.
- There is a risk of selection bias, as patients who feel strongly about AI or are able to speak English are more likely to volunteer to participate.
- Data collected in this small-scale feasibility study may be different from data collected in a large-scale real-world evaluation or routine care.

## Introduction

 Digital surgical wound monitoring, using a combination of patient-reported outcomes and images sent regularly to healthcare staff for review, is beginning to be implemented in many countries worldwide.^1^ Emerging evaluations are positive, with the key benefits including earlier diagnosis of surgical site infections (SSI), reduced use of unplanned National Health Service resources including general practitioner (GP) visits and potential for more targeted antibiotic use, which may support antimicrobial stewardship efforts.^2 3^ Patient satisfaction and engagement with digital surgical wound monitoring is high.^1 3 4^

However, implementing digital surgical wound monitoring, especially at scale, creates a new additional workload for staff and workforce solutions are required. A London-based Trust with the largest digital wound monitoring programme in the UK (covering an estimated 15 000 patients annually, across 10 surgical specialties at six hospitals) uses a centralised hub to streamline its staffing resource and provide a 7-day service for patients.^5^ Other emerging solutions involve artificial intelligence (AI). A small number of feasibility or exploratory studies are using AI to review patient-reported outcomes or wound images to predict SSIs or triage patients for urgent clinician review.^6–11^

Using AI within surgery is relatively new,^12^ and the success of digital interventions using AI is dependent on the acceptance of AI by end users; for digital surgical wound monitoring, the end users are staff and patients.^13 14^

Several studies and systematic reviews have explored patients and staff’s views of AI within healthcare. Overall, both patients and staff are supportive of AI, citing the potential for increased safety, access and efficiency but are concerned about digital security, staff over-reliance on AI, decreased safety and the loss of human interaction.^15–20^

However, no studies to date have explored patient and staff acceptability of the use of AI within digital wound monitoring. Thus, the aim of this study was to explore patient and staff acceptability of an AI-supported digital surgical wound monitoring platform, including perceptions of both the digital monitoring pathway and the use of AI to prioritise wound review.

## Materials and methods

### Study design

This qualitative study was embedded within the WISDOM randomised feasibility trial in which 120 patients at two cardiac hospitals in England were randomised to receive either AI-based digital surgical wound monitoring (called Isla) for 30 days after surgery plus usual wound care follow-up or usual wound care follow-up only.^3 6^ Patients in the intervention arm received weekly SMS text message requests to submit wound photos and information on healing. All submissions were reviewed, and they received a response from a healthcare worker. AI identified images with wound healing concerns and prioritised these for urgent clinician review. Usual care (UC) consisted of routine discharge advice, with patients instructed to contact their GP, community nurse or surgical team if they had concerns regarding wound healing. Interviews with a subset of 20 patients and 10 staff to explore acceptability of digital monitoring with AI were conducted between September 2024 and June 2025.

### Patient and public involvement

The study design and outcomes were reviewed by a Cardiovascular Lay Advisory Group. A patient and public involvement (PPI) representative with lived experience of cardiac surgery and surgical wound monitoring was a co-applicant on the study and led a PPI group that reviewed the interview guides and study processes and commented on interpretation of the findings. Findings were also discussed with study participants via a webinar.

### Setting, patients and eligibility

Two cardiac centres in England serving different patient populations were selected for the study. One centre had a wide geographical reach including rural and coastal areas, and the second centre had an inner-city location that served patients from a wider range of ethnicities. All adult patients undergoing coronary bypass artery graft (CABG) surgery were eligible to take part in the trial. Detailed inclusion and exclusion criteria are described elsewhere.^6^ Patients from the intervention group and the UC group were eligible to volunteer to participate in an interview. Although the UC group did not have direct experience of digital monitoring with AI, their views on the acceptability of AI were considered to be potentially informative. All clinical staff involved in delivering the feasibility study were eligible to participate in the staff interviews.

### Recruitment and consent

A subset of patient participants from the WISDOM feasibility study was recruited to take part in the interview. At the time of consenting to take part in the WISDOM study, all participants were given brief information about the optional interview and invited to state their willingness to be contacted to participate in the interview on their completion of the study. At study completion, delegated research nurses at the study sites purposively sampled 20 patients who had agreed to be approached to capture diversity by group allocation, sex, age, ethnicity and location. Information sheets and consent forms were provided by email at least 24 hours in advance of the interview. Consent was obtained at the start of the interview and was recorded and transcribed. Consent forms were signed by the interviewers.

All clinical staff involved in patient recruitment, digital wound review or outcome collection within the feasibility study were invited by the study site research nurses to participate in a staff interview. While sampling was planned to include staff from a range of sites and roles within the study, this was not carried out, as all staff who volunteered to participate were interviewed. Staff participants received information about the interview study in advance and provided informed consent prior to interview.

### Sample sizes

Interview participants represented a purposive subset of participants involved in the wider WISDOM feasibility trial. A target of 20 patients, around 10 from each group and 10 staff was planned. Data collection was guided by information power rather than reaching data saturation.^21^ Information power suggested that a relatively small sample would generate sufficient depth and relevance of data due to the focused study aim, use of an established theoretical framework and participants’ experience of the intervention.

### Interviews

Patient interviews were conducted after patients had completed their trial 30-day follow-up, and staff interviews were conducted towards the end of the trial. Staff and patient interviews were carried out by study site-delegated research nurses (KB, RM, KP and CM) who were provided with guidance and training from a member of the research team experienced in qualitative research (JT). Patient interviews were conducted remotely via Microsoft Teams, and staff interviews were conducted in-person at the participating sites using an interview guide. All interviews were digitally recorded and independently transcribed by a professional company and anonymised prior to analysis.

### Interview guide

Semi-structured interview guides were developed by the WISDOM research team, including PPI members. Questions explored participants’ experiences and perceptions of digital wound monitoring and AI, potential burden, implementation and adoption, surgical wound follow-up and experience of taking part in the study. Open-ended questions and prompts were used to allow participants to introduce issues important to them and explore emerging areas in greater depth. This paper focuses on data relating to digital monitoring and AI. Transcriptions of the first four interviews were reviewed, but no changes to the interview schedule or process were required to be made. See online supplemental material for the interview guides.

### Analysis

Analysis was conducted using a recursive process.^22^ Transcripts were read and re-read to support familiarisation with the data prior to coding by a member of the WISDOM research team with qualitative expertise (JT).^23^ Transcripts were initially coded deductively using the Theoretical Framework of Acceptability^24^ while allowing identification of inductive findings relevant to participants’ perceptions of digital wound monitoring and AI-supported care. The framework was selected because the study aimed to explore multiple dimensions of acceptability of AI-supported digital wound monitoring among patients and staff. Additionally, using a framework would minimise the influence of individual assumptions on analysis.

The Theoretical Framework of Acceptability^24^ includes the following seven constructs:

- Affective attitude: how patients and staff feel about the use of AI in digital wound monitoring
- Perceived effectiveness: the extent to which digital monitoring with AI is perceived as likely to achieve its purpose
- Ethicality: the extent to which digital monitoring and AI is a good fit with an individual’s or an organisation’s value system
- Intervention coherence: the extent to which patients and staff understand digital monitoring and AI and how it works
- Self-efficacy: patient and staff confidence that they can perform the behaviour required to participate in digital monitoring with AI
- Burden: the perceived amount of effort required to participate in digital monitoring with AI
- Opportunity costs: the extent to which benefits, profits or values must be given up or gained^25^ by engaging in digital monitoring with AI

Codes were subsequently grouped within the seven constructs and refined into subthemes representing shared patterns across participant accounts. Codes and interpretations were reviewed against the full dataset throughout analysis to ensure findings reflected the breadth and context of participant responses.

A second member of the WISDOM research team (MR) independently reviewed and coded an anonymised transcript using the Theoretical Framework of Acceptability to support trustworthiness and consistency of interpretation. Coding decisions, emerging interpretations and areas of uncertainty were discussed within the research team (including PPI representatives) and refined iteratively during the analytic process. Attention was also given to identifying views that differed from the dominant findings, including concerns regarding data security, overreliance on AI and loss of human interaction. Participants were invited to a webinar where findings were shared and discussed. Findings should be interpreted within the context of a qualitative feasibility study conducted in two cardiac surgery centres in England.

### Researcher characteristics and reflexivity

Interviews were undertaken by delegated research nurses at the study sites, who may have been known to participants through their involvement within the wider WISDOM study. These nurses had professional experience relevant to the study setting and patient group. KB and RM worked clinically on cardiac wards and units and were familiar with the management of SSI. KP and CM were research nurses working in cardiac surgery research and had experience supporting patients undergoing cardiac surgery and participating in research studies. The researcher nurses were familiar with postoperative wound care pathways, patient concerns following discharge and the practical delivery of digital wound monitoring. These experiences may have influenced how participants’ responses were understood and interpreted.^26 27^

Coding and analysis were led by the study academic lead and experienced qualitative researcher (JT). Analysis was checked by the study clinical lead (MR) who has extensive experience in surveillance, digital postoperative wound monitoring and implementation of remote monitoring pathways within NHS services. These professional backgrounds may have influenced how patterns and meanings within the data were recognised and interpreted, particularly regarding acceptability, implementation and the role of AI within routine postoperative care.^26 27^

Some researchers involved in the study had prior professional experience of digital wound monitoring and may therefore have held assumptions regarding its potential benefits for patients and healthcare services. Participants may also have viewed interviewers as being supportive of digital innovation, which may have influenced responses during interviews.^26^

## Findings

20 patient participants were interviewed (table 1); 12 patients from the Isla AI group and eight patients from the UC group. Fourteen patients were White, five patients were Asian or Asian British, and one patient did not state their ethnicity. Overall, 18 patients were male, and 17 patients lived in an urban setting. Although patients did not move between groups, one patient in the Isla AI group was unable to send information, and another patient did not receive any responses; one patient in the UC group was independently asked by a surgeon to send images of the wound through email. All 10 staff involved in conducting the study were interviewed (numbered 1–10); seven staff were from hospital A and three were from hospital B. Staff included nurses, research nurses and surgeons. Owing to the small numbers of staff involved, details are not provided to preserve anonymity. The median duration for the interviews was 19 (range 3–34) min.

**Table 1:** Participant demographics

Characteristic	Patients (n=20)
Age, median (range), years	65 (47–78)
Sex	
Male	18
Female	2
Group allocation	
Isla AI	12
Usual care	8
Ethnicity	
White, British and any other	14
Asian and Asian British	5
Not stated	1
Location	
Rural	3
Urban	17

Findings are presented within the seven constructs of the Theoretical Framework of Acceptability.

### Affective attitude

Both staff and patients were supportive of digital wound monitoring with AI and frequently described it as innovative and representative of future postoperative care.

I’m super-excited about the fact that you’re using AI to do this. Patient 7 Isla AI

I think the principle behind artificial intelligence, if it’s as good as a doctor or a nurse reviewing the pictures, then I think it’s an excellent idea. Staff 8 Hosp B

Several patients in the UC group reported disappointment at not being allocated to the AI enabled digital monitoring group.

So yeah, I was initially disappointed (to be allocated to the usual care groupto be allocated to the usual care group). It would have been nice to have been in the (AI digital monitoring) group. Patient 9 UC

A smaller number of both staff and patients expressed concerns regarding data security, distrust of AI and the possibility of over-reliance on technology without human oversight.

We have a lot of older clientele. Most of them are okay, but there are some of them that dislike technology, they don’t trust AI. The moment they hear the word AI they get disinterested and they don’t want to get involved. Staff 1 Hosp A

It felt a little unusual in the beginning, putting your trust in something that isn't physical, it’s not a person, it’s not a colleague. Staff 5 Hosp A

AI does have some famous or quite well-reported recent mistakes … but it isn’t actually operating on its own, is it? Patient 7 Isla AI

Some patients also wanted greater transparency regarding whether responses had been reviewed by clinicians or AI.

I’ll tell you the one thing that I found surprising, was the speed with which I got a reply. Telling me that my wound looked okay and was healing well and, because it was digital, I couldn’t tell whether a human being had looked at the photos…or whether, you know, an artificial intelligence platform had looked at it and went, mm, that looks good. Patient 5 Isla AI

I remember having a comment from somebody from the surgical team or somewhere…I’m not sure, so that wasn’t clear. So, that should have been clarified as to who is responding to my pictures. Patient 13 Isla AI

### Perceived effectiveness

Both staff and patients perceived digital wound monitoring with AI as effective for identifying wound concerns early and facilitating timely treatment.

We would be able to reduce the incidence of serious wound infection, because we’re identifying that really early and treating them. Staff 4 Hosp A

They picked it up quickly and managed it really quickly, which was good. Patient 15 Isla AI

Staff participants particularly emphasised the role of AI in prioritising patients for urgent review and supporting implementation of wound monitoring at scale.

It was flagging patients where they’d identified issues, and it was putting them at the top of the list to be reviewed. So those patients were getting seen quicker. Staff 5 Hosp A

It is quite impossible to face-to-face follow-up these big, large groups of patients on a regular basis. Staff 4 Hosp A

Staff participants also highlighted operational benefits including reduced workload, reduced readmissions and improved efficiency.

I think it will make the workload of the staff a lot easier. Staff 1 Hosp A

There are lots of patients that get admitted with wound infections … those patients will get reduced and that will free up some of the staff work so that they can engage in other areas. Staff 4 Hosp A

Patients and staff also highlighted reduced travel burden and easier access to specialist support, particularly for patients living far from surgical centres.

I think it’s a very efficient way to do wound surveillance because it’s easy for the patients. They don’t have to come in, especially if patients are living far away. Staff 1 Hosp A

It’s an easy way of getting information without dragging the patient up to the hospital. Patient 8 UC

Patients particularly valued rapid responses, reassurance and easier access to advice without needing GP appointments.

The advantage is somebody is going to look at it straightaway. So, you’ve got an instant answer response. Patient 6 Isla AI

Trying to get an appointment for a GP is never easy at the best of times. Patient 8 UC

Several participants felt earlier specialist review could support more timely treatment and potentially reduce delays in antibiotic management.

If the photos had worked properly, it could well have picked up the infection earlier, that made it easier for me to get antibiotics or sooner for me to get antibiotics which might have resulted in one dose of antibiotics being enough rather than two. Patient 20 Isla AI

Once we see the picture we can tell the patient, just come in, we’ll give you antibiotics or we’ll treat it accordingly. Staff 4 Hosp A

Both staff and patients valued the reassurance provided by the AI monitoring pathway.

It made me feel good that somebody was checking up and making sure that it was all good. Patient 15 Isla AI

I did feel that there was an extra eye on me, so I was more relaxed. Patient 2 Isla AI

You don’t know what’s right … it gives you a bit of peace of mind, doesn’t it, somebody’s checked it and looks okay? Patient 6 Isla AI

For us clinicians, it gave us assurance that we are providing appropriate and timely care to our patients. Staff 6 Hosp A

Staff participants also perceived that digital monitoring encouraged greater patient involvement in postoperative wound care.

It’s making them more involved in the post-op journey, so they’re actually monitoring the wound themselves and sending in the pictures. Staff 5 Hosp A

We’ve already experienced, from the feasibility study, that it also increases self-care of the patient. Staff 6 Hosp A

A small number of staff expressed concerns regarding the ability of the AI system to detect problems and assess wounds in patients with darker skin tones.

Sometimes Isla is flagging up that there is some colour change, but actually there is not, it is healing really well. Staff 4 Hosp A

Potentially darker skin … they don’t necessarily go red the way that other skin does. So it might be…that might be a bit of a tricky one to assess on a photo. Staff 9 Hosp B

Two patients in the digital monitoring group stated that they would still value some in-person surgical review in addition to digital monitoring.

It gives you a reassurance, having at least once, have a look at it proper. It is better if a surgeon has a look at it, like, one to one. Patient 4 Isla AI

I think I should have had at least one visit with the surgeon face to face just to look and make sure. Patient 6 Isla AI

### Ethicality

Staff participants appeared comfortable with the use of digital monitoring and AI within routine postoperative care and viewed the intervention positively.

I’m very excited to have this on board in our clinical practice. Staff 6 Hosp A

A lot of the staff that I’ve spoken to about the study have been very engaged and very interested in its development, so I think there’s a lot of expression of interest and people are open to adopting this kind of technology in practice. Staff 5 Hosp A

### Intervention coherence

Most staff and patients appeared to understand the purpose of the intervention and how the AI-supported monitoring pathway operated.

(AI and data security) does tend to cause them anxiety, but once you’ve given them the proper explanation, how the study works and how their personal information is safeguarded, that, kind of, eased everything. Staff 3 Hosp A

Why on earth wouldn’t this be a routine part of procedures in any health service? … it means that you’re still being cared for and you’re still being checked up on. Patient 5 Isla AI

### Self-efficacy

Both staff and patients generally found the digital monitoring platform straightforward and easy to use.

The technology that we use is quite straightforward, they don’t need to download an app, it’s very easy to use. Staff 1 Hosp A

Yes, that was quite simple really, you just took the photos and sent them. Patient 15 Isla AI

However, both staff and patients identified groups who might experience challenges using digital monitoring, particularly patients with limited digital access, lower digital confidence or poor English language proficiency.

People who don’t own mobile phones … could be severely disadvantaged if we were to adopt fully digital remote wound monitoring. Staff 1 Hosp A

There’s some people who are not tech savvy that may (struggle) but those people will always have someone around you. Patient 6 Isla AI

If it was in a language that they can understand, it would be a lot easier to get involved in the study. Staff 1 Hosp A

Older patients living alone were identified by staff as potentially requiring additional support.

The elderly might need more support. Staff 3 Hosp A

One staff participant questioned whether women may have different experiences of digital monitoring than men.

I would be interested on any feedback on whether female patients have a different experience to male patients. Staff 8 Hosp B

Most staff participants felt their hospitals were moving towards readiness to implement digital monitoring with AI, although several highlighted the need for training, staffing and IT integration.

I think we’re completely ready at the moment because we have trained the staff. Staff 4 Hosp A

It will take a lot of work integrating new software into the one that we’re already using … the staff would need to have training first. Staff 1 Hosp A

One nurse stated that the AI algorithm would need to be highly accurate before wider implementation.

Algorithm must be perfect before we roll out the system. Staff 7 Hosp A

### Burden

Both staff and patients felt participation in digital wound monitoring with AI was not burdensome.

It did not actually hamper my day-to-day work that much because it did not take too much time. Staff 4 Hosp A

It only really took about five or ten minutes every time there was a submission. Staff 9 Hosp B

It was quite easy to use, it only took a couple of minutes … it was really quick to use. Patient 14 Isla AI

However, staff participants anticipated that wider implementation at scale may require additional staffing support.

If this is going to roll out for every patient, it’s going to take a bit more doing … and probably a bigger team needed to review the wounds. Staff 9 Hosp B

### Opportunity costs

Staff and patient participants perceived that digital wound monitoring with AI could improve efficiency and reduce costs through reductions in travel, treatment burden and readmissions.

It’s more efficient … it saves everyone a lot of time as well. In the long term it will be more cost effective for everyone. Staff 1 Hosp A

Hospital admissions with serious wound infections will reduce and it will save the healthcare system a lot of money. Staff 4 Hosp A

Will be resource effective, time effective and cost effective of course. Patient 10 Isla AI

## Discussion

Although several systematic reviews of studies are focusing on patients’ and staff perceptions of AI within healthcare,^12 16 17 20^ this is among the first studies to explore patient and staff acceptability of AI within surgical wound monitoring.

Most importantly, patients and staff responded positively to all constructs of the Theoretical Framework of Acceptability.^24^ However, staff participants were directly involved in the feasibility study and findings may not reflect the views of wider clinical teams without prior exposure to digital wound monitoring or AI-supported pathways. Staff and patients believed the AI-based monitoring platform delivered what it set out to achieve; wound images with concerns were flagged for urgent review, and SSIs were identified early facilitating early treatment. One nurse stated the AI-based monitoring enabled regular monitoring to be implemented at scale, a service that could not be delivered using traditional surveillance methods.

Similar to the summaries of published reviews, both patients and staff in this study were generally supportive of using AI. As with other studies, participants felt safety was improved, access was increased and efficiency was improved. However, data security risks, staff over-reliance on AI and the loss of human interaction were common concerns.

In addition to the clinical benefits, perceived benefits to the NHS came from the reduced burden on staff resources and financial savings gained through reduced treatment and readmission costs. Participants also perceived that earlier specialist review of wound concerns may support more timely and targeted antibiotic treatment. Antibiotic prescribing and healthcare use were captured within the wider feasibility study and warrant further evaluation in future implementation research.^3^

In this study, patients immediately saw the potential benefits of regular wound monitoring and hoped to be randomised to the intervention group. The quick responses, increased convenience, easy access and low patient work burden likely led to their continued high levels of engagement.

Being reassured was important to patients who felt anxious about caring for their wounds at home. Feeling worried and a lack of support have been identified in previous studies.^28^ Having AI-based digital monitoring made patients in this study feel reassured, which appeared to have an impact on GP attendances. In the UK, UC advice for patients undergoing surgery at hospital discharge is to contact the GP for wound concerns. Digital surgical wound monitoring reduces unplanned GP visits,^2 3^ presumably from patients who have been reassured about their wound healing concerns.

A new finding from this study was that staff also felt reassured by the AI-based wound monitoring, as it could be implemented at scale, enabling all patients to receive appropriate and timely care. Surgical wound follow-up is resource intensive,^29^ and not all patients currently receive appropriate or timely surgical wound follow-up. An indication of the current scale of surgical wound surveillance can be seen in the 2025 report from the UK Health Security Agency where surveillance data are presented on only 146 411 operations out of an estimated 5 million.^30^

Interestingly, staff perceived that digital wound monitoring encouraged patient involvement in wound care, for example, by checking their wounds, keeping them clean for photos and submitting data, although patient behaviour change was not formally evaluated in this study.

Health equity is a key aim of the NHS 10-year plan.^31^ Several staff and patients suggested patients who were older, digitally disadvantaged or did not speak English might be negatively affected. Age, sex, race, geographical location, diagnosed disease, disease severity and level of education have all been found to influence patients’ attitudes towards AI.^16^ However, both staff and patients in this study said that most patients had someone, a carer or a family member, who could assist if needed and suggested that the only group of people who were truly adversely affected were patients with no support.

Data collected in this study shapes the following recommendations for the delivery of future AI-based surgical wound monitoring platforms.

- Patients were concerned about the loss of in-person contact. It is therefore important for some patients that digital wound monitoring runs in conjunction with, and not instead of, UC scheduled in-person appointments.
- Patients were concerned about staff over-reliance on AI decisions. It is suggested that AI-based monitoring should include clinician overview.
- Patients wanted transparency over whether activities were undertaken by clinicians or by AI. Therefore, text responses to patient submitted data should be signed by a clinician.
- Work is needed to increase engagement among potentially disadvantaged groups.
- While the burden of reviewing the small number of images in this feasibility study was not onerous, staff felt that the workload for reviewing a large number of images would require additional staffing. Using a centralised hub as part of UC to review submitted data would provide a sustainable solution.^5^

A limitation of the study was that participants were from two hospitals only, with most participants coming from just one hospital. Only two of the 20 patient participants were female, and none of the participants were black. This reflects national cardiac surgery surveillance data from the Health Security Agency, UK, which finds 20% of patients who underwent CABG are female, and approximately 2.1% are of black ethnicity.^30^ However, female patients and black patients have previously been identified as being less engaged with digital wound monitoring; thus, further research should be done with potentially disadvantaged groups.^32 33^

Staff participants were all involved in the delivery of the feasibility study, including patient recruitment, digital wound review or outcome collection and may therefore have been more familiar with, or supportive of, digital wound monitoring than staff not involved in the study. Patients and staff also self-selected to participate in the interviews, meaning those with stronger views, greater engagement with digital technology or more positive experiences of the intervention may have been more likely to participate. Findings should be interpreted with caution, as they may not reflect the views of all patients or staff involved in postoperative care.

The study was conducted during a small-scale feasibility study in which additional research support and training were available. Acceptability, workload and implementation challenges may differ during large-scale implementation within routine care settings. Wider implementation may require substantial organisational support, including staff training, pathway redesign, integration with existing IT infrastructure, allocation of staff time for image review and ongoing technical support. Therefore, the study may not have captured all barriers to implementation or resistance to adoption that could emerge during scale-up across wider clinical teams and organisations.

## Conclusion

Staff and patients were supportive of AI-based surgical wound monitoring. AI-based digital monitoring may help identify images with a wound healing concern and support earlier review of SSIs. Patients and staff described perceived benefits of AI-based digital monitoring in the WISDOM study, including easy access to the NHS, improved efficiency and cost savings. However, as with other studies, a small proportion of participants were concerned about data security, staff over-reliance on AI decisions and the loss of human interaction. The results suggest that AI-based digital monitoring may have potential to support wider implementation.

## Supplementary material

10.1136/bmjopen-2026-116323 online supplemental file 1

10.1136/bmjopen-2026-116323 online supplemental file 2

## Data Availability

Data are available upon reasonable request.
