# Supplementary material for: Exploring patient and NHS staff acceptability of artificial intelligence-supported surgical wound monitoring within the WISDOM feasibility study: a multi-centre qualitative interview study
Source: BMJ Open. 2026 Jul 31;16(7):e116323. doi: 10.1136/bmjopen-2026-116323 (PMC13436100; doi:10.1136/bmjopen-2026-116323)
Supplement: Supplementary file 1 [file bmjopen-2026-116323.reviewer_comments.pdf]

PEER REVIEW HISTORY

BMJ Open publishes all reviews undertaken for accepted manuscripts. Reviewers are asked to complete a checklist review form and are provided with free text boxes to elaborate on their assessment. These free text comments are reproduced below.

ARTICLE DETAILS

Title (Provisional)

Exploring patient and NHS staff acceptability of artificial intelligence (AI)-supported surgical wound monitoring within the WISDOM feasibility study: a multi-centre qualitative interview study

Authors

Tanner, Judith; Rochon, Melissa; Cariaga, Karen; Harris, Roy; Beckhelling, Jacqueline; Bouttell, Janet; Bolton, Sarah; WILSON, KEITH; Jurkiewicz, James; Dhoonmoon, Luxmi; Mostafa, Nada; Dummer, Jon; Basilio, Kristia; Magboo, Rosalie; Procter, Kathryn; McMillan, Claire

---

VERSION 1 - REVIEW

**Reviewer**	**1**
**Name**	**Ousey, Karen**
**Affiliation**	**University of Huddersfield, Human and Health Sciences**
**Date**	**23-Feb-2026**
**COI**	**I am currently chairing a steering committee for a research study that is being led by Professor Tanner and Melissa Rochon.**

Thank you got asking me to review this manuscript. I believe the readers will find it of interest. AI is becoming more prevalent in clinical areas and is gaining much discussion. Within SSI I believe the use of AI will provide early identification of potential wound infection - as you have noted in this paper. I was interested to note that none of the participants were under 40 and all managed to use the system - over the next few years I am sure the use of AI for surveillance will become the norm. I see one of your limitations was the limited number of females and those with Black skin tone - are you able to offer a short discussion of why this was e.g., were these 2 groups approached and refused? You mention there is a cost saving with early identification of wound infection - I think the paper would benefit from some more detailed re: potential savings and I would like to see mention of AMR and how AI monitoring can potentially reduce the risk of AMR and become a part of the AMS toolkit. Overall I found the paper interesting

---

**Reviewer**	**2**

Name **Akinwusi, Olukunle**

Affiliation **Digital Health, Foundation for Innovative New Diagnostics, Geneva, Switzerland Chemin du Pommier 40 1218 Grand-Saconnex, Switzerland**

Date **27-Mar-2026**

COI **None**

Wonderful write up and the methods are generally clear. However, they are not yet sufficiently detailed for replication. The manuscript would benefit from fuller reporting of the interview guide/content, sampling logic across intervention versus usual-care participants and clearer description of how themes/interpretations were developed from the Theoretical Framework of Acceptability. Also, admittably, the study lIimitations are acknowledged, including the small number of sites and under-representation of women and Black patients, but the limitations section could fully address more, such as potential selection bias.

Reviewer **3**

Name **Hoffmann, Christin**

Affiliation **University of Bristol, Population Health Sciences**

Date **23-Apr-2026**

COI **None**

This manuscript is well written, with some areas that could be improved.

Major points

1. Throughout the first part of the manuscript (particularly methods), lines seems to be blurred between the larger study and this qualitative study. I think the manuscript would be clearer if the focus is kept on the latter, the actual study presented here. E.g. the PPI section talks about surveys, the recruitment section seems to outline the patient recruitment process for the trial, rather than how people were recruited to this qualitative study. At the moment, I read it as if every trial participant also completed an interview, but I assume it would be a sub-set rather? Similarly, only 10 professionals were interviewed and there is information about their eligibility and that these were invited, but there is less detailed information about the recruitment and consenting process and doesn't quite fulfil the criteria in the SRQR checklist.

2. My second major point related to reporting deficiencies that impact transparency and judgements about the methodological rigour. I would urge the authors to carefully review the SRQR as a lot of the required reporting items are not found in the manuscript. These are important to ensure the paper is of certain quality and results can be trusted. Atm, I have no

confidence, simply because there are some essential methodological details missing. Some outlined below, but please review carefully:

a. I am missing a description of the sampling criteria. I appreciate the authors refer to a different publication to get detailed information about eligibility criteria, but I wondered if sampling strategy was at all informed by patient/professional characteristics.

b. Please provide a bit more detail about the "research staff" in the "interview" paragraph, particularly whether "research staff" is academic, clinical or otherwise? And as written atm, I am unsure if they were trained in qualitative methods as it states they were only offered the training. Please refer back to the checklist to ensure all information in the "Researcher characteristics and reflexivity" are described.

c. Please include the two topic guides in the appendices

d. Authors state "most interviews lasted around 20min". In all qualitative studies, I would expect the mean and range to be reported. So please include this information if you can.

e. There is insufficient detail in the "Analysis" section and some basic information is missing to understand the methodological rigour of analysis. For example, the underlying analytical approach isn't stated or referenced. I assume framework analysis and deductive coding was used? Who performed the coding and how was data triangulated or what other quality assurance processes were in place? Again, all the necessary reporting items are outlined in the SRQR.

f. Why was a framework chosen to conduct the analysis rather than selecting more inductive and explorative approaches? I think it would be helpful for readers to explain the framework and detail the categories in the methods. The results are structured accordingly and it is essential to understand the conceptual meaning of those categories to be able to make sense of the findings.

Minor points

1. The focus of the study that needs a little bit of clarification, mainly because the last two sentences of the introduction suggest a minor but important distinction. Is the focus of the study the use of AI specifically, or the use of the platform in general that includes AI? The majority of the findings seem to particularly relate to AI, whereas some are only applicable to the platform itself (e.g. "using digital wound monitoring was not onerous.").

2. The findings section could be better structured to improve clarity. Reporting within each category can be clearer by presenting findings by which ones are related to staff only, which are related to patients only, and which findings cut across both participant groups.. and keep this structure consistent for all framework categories.

3. I think it would be important to state somewhere what usual care is to understand what perspective those patients are taking.

4. There are some queries related to the results content:

a. The last section in affective attitudes “The speed with which one patient received responses” etc: I don’t understand why this isn’t coded as perceived effectiveness?

b. I am unsure what “The regular weekly review” refers to. I think a bit of context is required to understand that finding

c. The second category contains a lot of information with the third main point containing a large amount of information and exemplary queries over 3 pages. I think this could be heavily condensed.

d. I am surprised to see no evidence from patients in the opportunity cost section, but rather some “leftover evidence” that wasn’t included in the effectiveness category. I don’t think it is helpful if the categories overlap in their conceptualisation. I would expect the cost saving benefits to be included in this last section. But it may depend how you defined effectiveness of the intervention, which is why, again, I think some explanation about the categories further up would be helpful.

e. The finding “One nurse observed that fewer women were recruited to the study than men, suggesting a possible gender divide.” Is not related to the platform and shouldn’t be in the results. That is a study limitation.

f. I think the results are framed very positively with relatively few concerns/barriers to acceptability listed, particularly amongst staff. I think it is important to discuss that the entire staff sample consisted of only staff included in the roll-out of the monitoring platform. The results may as well differ amongst those not already using the platform. This is especially important because of the emphasis on aiming for a large scale roll out. For example, the exemplary quote in the finding that “at an organisational level, their hospitals were ready to implement digital monitoring with AI” suggests that there is a process of training involved. And training in and of itself is a significant operational element and time burden, let alone the IT infrastructure. I think the limitations section should be extended to discuss that the timing of the study and self-selected staff sample potentially omits identification of acceptability issues. For readers wanting to implement a system like this at scale where none exist, it would be useful to anticipate barriers related to all the necessary steps involved in onboarding a system like, before it runs smoothly within a team environment, because there always are realistic difficulties.

5. In the discussion, I think the sentence “This behavioural change aligns with initiatives to promote patient selfcare and paves the way for supported patient self-care activities” is overstated as the study didn’t collect evidence on actual behaviour change. It is also interesting that this was identified in staff interviews, but it is not a finding that seems replicated in actual patient interviews. So, I do question the strength of the evidence here.

6. I don’t understand the meaning of the sentence “Implementation at scale was the driving motivation for AI-based wound monitoring”. It feels out of place and not grounded in evidence.

7. The setting described in the Abstract contains additional detail, i.e. hospitals in England. I think this should be stated in the main body too.

VERSION 1 - AUTHOR RESPONSE

Comments	Responses
Reviewer 1	
I see one of your limitations was the limited number of females and those with Black skin tone - are you able to offer a short discussion of why this was e.g., were these 2 groups approached and refused? You mention there is a cost saving with early identification of wound infection - I think the paper would benefit from some more detailed re: potential savings and I would like to see mention of AMR and how AI monitoring can potentially reduce the risk of AMR and become a part of the AMS toolkit. Overall I found the paper interesting	Thank you. We have discussed national data for cardiac surgery (sex and ethnicity) in the limitation section.<br><br>We have added information about antibiotics to the intro, results and discussion. And in the discussion we refer to the feasibility study findings where the costs are discussed
Reviewer 2	
Wonderful write up and the methods are generally clear. However, they are not yet sufficiently detailed for replication. The manuscript would benefit from fuller reporting of the interview guide/content, sampling logic across intervention versus usual-care participants and clearer description of how themes/interpretations were developed from the Theoretical Framework of Acceptability. Also, admittedly, the study limitations are acknowledged, including the small number of sites and under-representation of women and Black patients, but the limitations section could fully address more, such as potential selection bias.	Thank you for your review. We have; added interview guides as Supplemental Material; clarified sampling logic and provided a more detailed description of how themes/interpretations were developed from TFA; and discussed selection bias in limitations
Reviewer 3	
1. Throughout the first part of the manuscript (particularly methods), lines seems to be blurred between the larger study and this qualitative study. I think the manuscript would be clearer if the focus is kept on the latter, the actual study presented here. E.g. the PPI section talks about surveys, the recruitment section seems to outline the patient recruitment process for the trial, rather than how people were recruited to this qualitative study. At the moment, I read it as if every trial participant also completed	Thank you. In response to your comments, we revised the Methods section to clarify that this was a qualitative sub study embedded within the feasibility trial. We have removed mentions of the main WISDOM study where

an interview, but I assume it would be a sub-set rather? Similarly, only 10 professionals were interviewed and there is information about their eligibility and that these were invited, but there is less detailed information about the recruitment and consenting process and doesn't quite fulfil the criteria in the SRQR checklist.	possible. We have rewritten the recruitment section.
2a. I am missing a description of the sampling criteria. I appreciate the authors refer to a different publication to get detailed information about eligibility criteria, but I wondered if sampling strategy was at all informed by patient/professional characteristics.	This information has been added.
2b. Please provide a bit more detail about the "research staff" in the "interview" paragraph, particularly whether "research staff" is academic, clinical or otherwise? And as written atm, I am unsure if they were trained in qualitative methods as it states they were only offered the training. Please refer back to the checklist to ensure all information in the "Researcher characteristics and reflexivity" are described.	We have clarified that study site research nurses were provided training and added a new section on Reflexivity where we describe the research site staff in more detail.
2c. Please include the two topic guides in the appendices.	Added as Supplemental Material.
2d. Authors state "most interviews lasted around 20min". In all qualitative studies, I would expect the mean and range to be reported. So please include this information if you can.	Added to the findings
2e. There is insufficient detail in the "Analysis" section and some basic information is missing to understand the methodological rigour of analysis. For example, the underlying analytical approach isn't stated or referenced. I assume framework analysis and deductive coding was used? Who performed the coding and how was data triangulated or what other quality assurance processes were in place? Again, all the necessary reporting items are outlined in the SRQR.	Additional information added.
2f. Why was a framework chosen to conduct the analysis rather than selecting more inductive and explorative approaches? I think it would be helpful for readers to explain the framework and detail the categories in the methods. The results are structured accordingly and it is essential to understand the conceptual meaning of those categories to be able to make sense of the findings.	We have added a rationale for using the Theoretical Framework of Acceptability (TFA) and moved the information about the categories from the findings to methods.
1. The focus of the study that needs a little bit of clarification, mainly because the last two sentences of the introduction suggest a minor but important distinction. Is the focus of the study the use of AI specifically, or the use of the platform in general that includes AI? The majority of the findings seem to particularly relate to AI, whereas some are	We have clarified the aim of the study in last sentence in the introduction and have amended the findings where appropriate.

only applicable to the platform itself (e.g. “using digital wound monitoring was not onerous.”).	.
2. The findings section could be better structured to improve clarity. Reporting within each category can be clearer by presenting findings by which ones are related to staff only, which are related to patients only, and which findings cut across both participant groups. and keep this structure consistent for all framework categories.	Thank you. We tried separating out staff and patient responses but that did not work well. Instead, we improved the signposting throughout, and we re-wrote most of the ‘perceived effectiveness’ section which, on reflection, was hard to follow.
3. I think it would be important to state somewhere what usual care is to understand what perspective those patients are taking.	Added to the study design section.
4. There are some queries related to the results content:<br>a. The last section in affective attitudes “The speed with which one patient received responses” etc: I don’t understand why this isn’t coded as perceived effectiveness?	We have amended this text to make it clearer that the patient was concerned about transparency of what work the AI was doing – i.e. was the speedy response written by a nurse or was it AI generated
4b. I am unsure what “The regular weekly review” refers to. I think a bit of context is required to understand that finding.	We have added information to the study design section describing the digital monitoring with AI (which includes a weekly review) and usual care.
4c. The second category contains a lot of information with the third main point containing a large amount of information and exemplary queries over 3 pages. I think this could be heavily condensed.	We have condensed this section
4d. I am surprised to see no evidence from patients in the opportunity cost section, but rather some “leftover evidence” that wasn’t included in the effectiveness category. I don’t think it is helpful if the categories overlap in their conceptualisation. I would expect the cost saving benefits to be included in this last section. But it may depend how you defined effectiveness of the intervention, which is why, again, I think some explanation about the categories further up would be helpful.	Only one patient mentioned cost savings and we have included this quote in the cost section.<br><br>It was not possible to remove the quotes on efficiency and cost savings from the perceived effectiveness section as these are perceived attributes of digital monitoring and AI.

	We have removed the explanation of the categories from the findings section and included them further up within the description of the Framework.
4e. The finding "One nurse observed that fewer women were recruited to the study than men, suggesting a possible gender divide." Is not related to the platform and shouldn't be in the results. That is a study limitation.	This quote has been removed, and the low number of female participants is discussed in the limitation section.
f. I think the results are framed very positively with relatively few concerns/barriers to acceptability listed, particularly amongst staff. I think it is important to discuss that the entire staff sample consisted of only staff included in the roll-out of the monitoring platform. The results may as well differ amongst those not already using the platform. This is especially important because of the emphasis on aiming for a large scale roll out. For example, the exemplary quote in the finding that "at an organisational level, their hospitals were ready to implement digital monitoring with AI" suggests that there is a process of training involved. And training in and of itself is a significant operational element and time burden, let alone the IT infrastructure. I think the limitations section should be extended to discuss that the timing of the study and self-selected staff sample potentially omits identification of acceptability issues. For readers wanting to implement a system like this at scale where none exist, it would be useful to anticipate barriers related to all the necessary steps involved in onboarding a system like, before it runs smoothly within a team environment, because there always are realistic difficulties.	Thank you for this helpful comment. In response, we have revised the abstract, discussion, limitation and conclusion sections to provide a more balanced interpretation of the findings.
5. In the discussion, I think the sentence "This behavioural change aligns with initiatives to promote patient selfcare and paves the way for supported patient self-care activities" is overstated as the study didn't collect evidence on actual behaviour change. It is also interesting that this was identified in staff interviews, but it is not a finding that seems replicated in actual patient interviews. So, I do question the strength of the evidence here.	We have amended.
6. I don't understand the meaning of the sentence "Implementation at scale was the driving motivation for AI-	We have deleted the sentence.

based wound monitoring". It feels out of place and not grounded in evidence.	
7. The setting described in the Abstract contains additional detail, i.e. hospitals in England. I think this should be stated in the main body too.	Added

VERSION 2 - REVIEW

**Reviewer**	**3**
**Name**	**Hoffmann, Christin**
**Affiliation**	**University of Bristol, Population Health Sciences**
**Date**	**13-Jul-2026**
**COI**	

Thank you for addressing my comments, and I have no further comments. It would have been helpful to see the actual changes made in your response document. Without the use of track changes or copy/pasting of sections that are relevant to the individual reviewer comments, it is difficult to judge whether comments have, in parts, been adequately addressed.
