# Supplementary material for: Exploring patient and NHS staff acceptability of artificial intelligence-supported surgical wound monitoring within the WISDOM feasibility study: a multi-centre qualitative interview study
Source: BMJ Open. 2026 Jul 31;16(7):e116323. doi: 10.1136/bmjopen-2026-116323 (PMC13436100; doi:10.1136/bmjopen-2026-116323)
Supplement: Supplementary file 2 [file bmjopen-16-7-s001.docx]

**WISDOM semi-structured interview guide**

**What were your experiences of taking part in the WISDOM study?** (focusing on the recruitment process, information they received, motivation for taking part, willingness to be randomised, preferred methods of dissemination, any improvements that could be made).

***What were your experiences of using Isla digital monitoring?** (focusing on their ability to use technology, to take photos, the frequency and duration of monitoring, their access to NHS support, limiting or challenging issues, any advantages or disadvantages)

**What was your experience of wound follow-up after being discharged?** (how they felt regarding support, interactions with the NHS, ease of access to NHS support, satisfaction with service provided, benefits or drawbacks)

**How are you feeling now after your surgery?** (experience of wound infections or complications, impact on daily living activities)

*Only for Isla participants
