# Supplementary material for: Exploring patient and NHS staff acceptability of artificial intelligence-supported surgical wound monitoring within the WISDOM feasibility study: a multi-centre qualitative interview study
Source: BMJ Open. 2026 Jul 31;16(7):e116323. doi: 10.1136/bmjopen-2026-116323 (PMC13436100; doi:10.1136/bmjopen-2026-116323)
Supplement: Supplementary file 3 [file bmjopen-16-7-s002.docx]

**WISDOM Semi-structured interview schedule - STAFF**

**Involvement in the study**

Were you involved in the WISDOM study?

Did you feel you were well prepared for the study?

What, if anything could have improved your experience?

**Experience of wound monitoring as part of the study**

What was your involvement in the study?

How did the study impact on your work?

How much time did it take up?

**Opinions of digital surgical wound monitoring and artificial intelligence**

What are your thoughts on using digital wound monitoring?

What are your thoughts on using artificial intelligence to support digital wound monitoring?

What challenges or concerns do you think might affect adopting remote wound monitoring at your hospital?

Are there specific patient populations for which remote wound monitoring might be more challenging or less effective?

How comfortable are you with the integration of image-based technology into wound monitoring practices?

What types of support or training would you need to effectively implement remote wound monitoring?

What role do you think artificial intelligence might play in remote wound monitoring that would be useful for staff?

How do you think remote wound monitoring might impact patient engagement and self-care?

How concerned are you about the security and privacy of patient data in the context of remote wound monitoring?

What measures would you like to see in place to address these concerns?

How ready do you feel your hospital is to implement remote wound monitoring?

What factors would contribute to the successful implementation of remote wound monitoring in your healthcare setting?

What improvements or new features you would like to see in future remote wound monitoring technologies? open-ended,

Where do you see this technology in five years time'?
